# The association between socioeconomic status and C-reactive protein in Bayesian perspective

**DOI:** 10.1016/j.ssmph.2023.101464

**Published:** 2023-07-20

**Authors:** Alessandro Procopio, Robin Samuel

**Affiliations:** a Stockholm University, Sweden; b University of Luxembourg, Luxembourg

## Abstract

• This study analyzed the total health inequality on risks of chronic inflammation.
• We used Bayesian multiple and distributional regression models.
• Between-group posterior distributions show a robust educational gradient in health.
• Within-group posterior distributions show polarized risks for individuals.

## 1 Introduction

The graded association between socioeconomic status (SES) and mortality risks is an established finding in the biomedical and social scientific literature (Adler & Ostrove, 1999; Brummett et al., 2014; Harris & Schorpp, 2018; House, 2002; Winkleby et al., 1992; Yang et al., 2020). While there is a broad literature that links individuals’ social conditions and health outcomes (Adler et al., 1994; Adler & Ostrove, 1999; Clark et al., 2009; Elo, 2009; Ferraro & Shippee, 2009; Marmot et al., 1991; Pudrovska, 2014), the focus on mechanisms driving this relationship is more recent (Elo, 2009; Freese, 2018; Harris & Schorpp, 2018). Particularly, social scientists and epidemiologists have been focusing on the mediatory role of chronic illness and inflammation on the association between individuals’ SES and health disparities (Baum et al., 1999; Dowd & Zajacova, 2007; Pudrovska, 2014) in their life course (Ben-Shlomo & Kuh, 2002; Corna, 2013; Ferraro & Shippee, 2009; Hallqvist et al., 2004; Liu et al., 2017; Power et al., 1999; Yang et al., 2020). Although previous studies have focused on measuring the social group differences in health outcomes (Kawachi et al., 2002), little is known regarding the differences among individuals belonging to those social groups. Individual variation within social groups has been significantly understudied compared to the between-group analysis. Within the World Health Organization (WHO), public health scientists have engaged in a heated debate concerning the use of an individual-level approach (Gakidou et al., 2000; Murray et al., 1999), or a group-level comparison strategy (Braveman et al., 2000). After the 2000s, social scientific and public health literature primarily focused on “between group” comparisons (across countries, ethnicities, gender, or social conditions). The within-group aspect of health distribution regained interest among social and public health scientists in the total health inequality theory context (Gakidou & King, 2002). Particularly, the total health inequality theory reframes the previous debate within the WHO where the within and between group comparisons are complementing – not competing – components of inequality. This study investigates how individuals’ SES shapes the distribution of C-reactive protein (CRP). We address the following research questions: How does individuals’ SES shape the distribution of C-reactive protein (CRP)? How is the CRP distribution shaped within SES categories? Our main aim is to explore these questions without testing specific hypotheses. CRP is an acute-phase protein produced by hepatocytes as a response of the immune system to acute infection or systemic inflammation. Biomedical studies linked CRP to mortality risks due to cardiovascular diseases (CVDs, Alley et al., 2006; Harris et al., 1999; Laaksonen et al., 2005; McDade et al., 2011). CVDs are one of the leading causes of mortality and morbidity in high-income countries (Brummett et al., 2014; Liu et al., 2017; Mitchell & Aneshensel, 2017). Previous studies have found evidence concerning the socially patterned onset of CVDs, where individuals from a lower SES encountered higher risks of mortality due to CVDs (Goodman et al., 2005; Lubbock et al., 2005; Winkleby et al., 1992), confirming the social causation theory (Link & Phelan, 1995; Phelan et al., 2010). Additionally, CVDs cause a considerable burden on individual and public health. Therefore, biomedical and social science research has striven to understand and identify the principal physiological changes that could signal the onset of CVDs in individuals at the pre-symptom stage of the disease (Davillas et al., 2019; Dowd & Zajacova, 2007; McEwen, 2015; Mitchell & Aneshensel, 2017). Prevention and identification of the population at significant risk of CVDs have been a primary objective for social scientists, public health policy-makers, and epidemiologists (Davillas et al., 2017; Herd et al., 2007; Mitchell & Aneshensel, 2017). In the field of social inequalities in health, accumulating evidence highlights the association between CRP and individuals’ social conditions, where individuals in higher SES position have lower levels of CRP, and likely, it might lower the risks of CVDs onset (Brummett et al., 2014; Davillas et al., 2019; Gimeno et al., 2008; Jousilahti et al., 2003; Karimi et al., 2019; Koster et al., 2006; Lubbock et al., 2005; McDade et al., 2011). Empirical evidence has highlighted the importance of health-related behaviors, such as smoking, sedentary lifestyle, and alcohol consumption, as mediating factors in the social gradient of CRP. In particular, previous studies have shown that individuals in lower social conditions are more prone to engaging in health-risky behaviors, thereby linking SES to CRP levels (Alley et al., 2006; Yang et al., 2020). Additionally, recent studies have demonstrated empirically an interplay between race and SES (Farmer et al., 2022, Farmer et al., 2020, Farmer et al., 2021).

Our study contributes in two ways to this emergent research strand by first investigating how the social determinants of health get under the skin, addressing the mechanism linking individuals’ SES with CRP levels. Second, this study proposes the Bayesian paradigm as an alternative methodological framework to measure the total inequality in CRP, which is our individuals’ health indicator. We employed two types of Bayesian Regression Model (BRM) to enhance our understanding of the social determinants of health. Using the BRM, our focus of the empirical analysis shifts from the conventional point estimates (and their statistical significance) to a distribution of likely parameters, allowing the detection of a more comprehensive understanding of the potential impacts that individuals’ social conditions exert on their health and well-being. We expanded the analysis range by exploring the intra-group differences in CRP levels between individuals at the same SES level. Consequently, we modeled the standard deviation of CRP distribution as a function of individuals’ SES through a Bayesian distributional model (Umlauf & Kneib, 2018). Our study investigates the mediating role of health-related behaviors, focusing on smoking status, physical activity, and Body Mass Index (BMI), and their risk factors. We used data from the wave 2012 (Health Assessment Panel) of the United Kingdom Household Longitudinal Study (UKHLS), a nationally representative longitudinal survey set in the United Kingdom^2^.

To consider the multidimensional characteristics of SES, we included three measures of individuals’ social conditions in the empirical analysis: occupational status, educational level, and household income (see Elo, 2009; Goldthorpe, 2010 for a methodological discussion).

## 2 Data & methods

### 2.1 Data

The data we used for the empirical analysis comes from the UK Household Longitudinal Study (UKHLS), wave 2 (Health Assessment Panel, 2012). The Understanding Society – UKHLS is a large and representative survey of households sampled in the United Kingdom (UK), Scotland, Wales, and Northern Ireland. Administration of the main survey interested the General Population survey (GPS)^3^.

The sampling procedure for the GPS consisted of a two-stage step: the first primary sampling unit (PSU) consisted of a sample of postcode sectors, within which the addresses were the sampling units. The UKHLS provides a multi-purpose questionnaire to the respondents, which covers various topics relevant to social research. In 2010 and 2012, alongside the main questionnaire, the survey design included questions on health, and it collected blood samples from the respondents who consented to participate. The eligibility criteria for the respondents to participate in the nurse health assessment included completing the face-to-face interview; being 16 or older; living in England, Scotland, or Wales; completing the questionnaire in English, and not being pregnant (Mcfall et al., 2012). Individuals with HIV, hepatitis A or B, and clotting or bleeding disorders were excluded. The aim of collecting biospecimens by registered nurses was to gather information on potential health risks. Furthermore, blood sample collection supports genetic analyses and creates a genetic database. The nurse health assessment interested a subsample of the GPS and included anthropometric measures (such as height, weight, percent body fat, and waist circumference), blood pressure, grip strength, lung function, and blood samples. The basis of the biomarkers selection from the blood samples considers the environmental effect (socioeconomic, physical, or psychosocial), the impact on the biospecimen, its importance to essential health conditions, and the proportion of the population affected by the disease. From the 9896 observations of the initial sample, 521 individuals recorded a missing value of CRP. Thus, we excluded those cases from the study. Individuals with inapplicable values on height and weight measures to calculate individuals’ BMI (N = 283) were set as missing values and excluded from the statistical analysis. After deleting missing values on the covariates, the size of our analytical sample was N = 8,960^4^. We conducted a subgroup analysis by testing the difference in observable values of the covariates included in the model. Appendix B provides a summary statistics table on the characteristics of the sample having missing values and results of statistical significance tests between the analytical sample and the missing cases on covariates in models 1 and 2.

### 2.2 Measures

#### 2.2.1 Dependent variable

The dependent variable of the two BRMs is the recorded CRP in the Wave 2012 of the Understanding Society - UKHLS, measured in mg/L. We considered high-risk CVDs individuals with a CRP level greater than 3 mg/L, which is the cut-off point for defining an individual affected by low-grade inflammation (Brummett et al., 2014; McDade et al., 2011). As the distribution of the recorded values among the individuals in the sample is highly skewed, we log-transformed the variable to normalize the distribution.

#### 2.2.2 Covariates

##### 2.2.2.1 SES measures:

To capture the multidimensionality of SES (Elo, 2009; Goldthorpe, 2010), we included in the statistical model three measures of SES: occupational status, education, and income. Occupational status comprises eight categories from the National Statistics Socioeconomic Classification (NS-SEC): large employers & higher management, higher professional, lower management & professional, intermediate, small employers & own account, lower supervisory & technical, semi-routine, routine, and not in LM. The last category includes retired individuals, students, and individuals that are not currently working. The main reason is to compare individuals who have a job (and within those, compare the occupational categories) with individuals who do not currently have a job.

Educational level was measured using five categories: degree, other higher degree, A-level, GCSE, other qualifications, and no qualification.

We have taken the gross household income that was registered the month before the interview concerning the third SES measure. Then, we equivalized the scale, dividing the income scale by the equivalence scale set by the OECD, returning the equivalized income scale for the number of household members.

##### 2.2.2.2 Mediating factors:

The health behavior of individuals that might influence CRP levels (Dowd et al., 2009; Yang et al., 2020) has been measured by taking into account the level of sports activity (scale from 0 “no activity” to 10 “very active”), the Body Mass Index (BMI) calculated as weight/(height/100)^2^, smoking behavior (current smoker, ex-smoker and non-smoker), and self-rated health (as it is associated with CRP levels, see Shanahan et al., 2014), which have been coded into five categories: excellent, very good, good, fair, and poor health.

##### 2.2.2.3 Controllers:

The covariates we included to control the relationship between the SES measures and the CRP levels concern the individuals’ sociodemographic characteristics.Among the sociodemographic variables, for the analysis, we considered the age (measured as a continuous scale from 20 to 65 years old) of the individuals, gender, and house ownership (see McDade et al., 2011) as indicator of wealth.

### 2.3 Descriptive statistics

Table 1 provides an overview of the descriptive statistics (mean and standard deviations) of the covariates included in the models, while Table 2 provides the correlation matrix among the variables included in the analysis of Understanding Society data.

**Table 1:** Summary Statistics of the dependent variable and the covariates. Age, Income, BMI, and Sport Activity are *z*-standardized for computational efficiency of the MCMC algorithm.

Variables	Mean	St. Dev
CRP (log scale)	0.444	1.093
Sport Activity	3.398	2.896
Age	52.069	16.713
Income	1978.723	1534.189
BMI	27.960	5.096
**Occupation**		
Large employers & higher management	0.032	0.176
Higher Professionals	0.044	0.205
Lower management & professional	0.162	0.369
Intermediate	0.079	0.269
Small employers & own account	0.055	0.228
Lower supervisory & technical	0.044	0.204
Semi routine	0.095	0.293
Routine	0.055	0.228
Not in LM	0.435	0.496
**Education**		
Degree	0.221	0.415
Other higher degree	0.137	0.344
A-level etc	0.182	0.386
GCSE etc	0.197	0.398
Other qualification	0.117	0.322
No qualification	0.145	0.353
**Gender**		
Male	0.441	0.497
Female	0.559	0.497
**House Ownership**		
Owned	0.762	0.426
Rent	0.225	0.418
Other	0.013	0.112
**Self-rated Heath**		
Excellent	0.154	0.361
Very good	0.355	0.478
Good	0.287	0.452
Fair	0.154	0.361
Poor	0.050	0.219
**Smoking Behavior**		
Smoker	0.197	0.398
Ex smoker	0.403	0.491
Non smoker	0.400	0.490

**Table 2:** Correlation matrix of the covariates included in the analysis of Understanding Society data.

	CRP	Occupation	Education	Gender	House Ownership	SRH	Smoking	Sport Activity	Age	Income
CRP										
Occupation	0.15***									
Education	0.18***	0.44***								
Gender	0.07***	0.05***	0.05***							
House Ownership	0.09***	0.14***	0.16***	0.04***						
Srh	0.22***	0.27***	0.26***	0.00	0.15***					
Smoking	−0.09***	−0.09***	−0.15***	0.07***	−0.21***	−0.17***				
Sport Activity	−0.22***	−0.22***	−0.25***	−0.11***	−0.09***	−0.37***	0.10***			
Age	0.16***	0.39***	0.28***	−0.04***	−0.17***	0.21***	0.04***	−0.30***		
Income	−0.12***	−0.41***	−0.33***	−0.05***	−0.18***	−0.20***	0.11***	0.17***	−0.11***	
BMI	0.37***	0.06***	0.12***	0.00	0.06***	0.23***	0.02*	−0.19***	0.12***	−0.08***

### 2.4 Statistical analysis

The empirical analysis provided two Bayesian regression models to draw a posterior distribution from all the possible magnitude effects through Markov Chain Monte Carlo (MCMC) algorithm (Lynch & Bartlett, 2019). In the context of the Bayesian linear regression model, the posterior distribution of the parameters *β*_0_ (the intercept), *β*_1_ (the slope of the regression lines), and *σ* (the standard deviation) are:

(1) $$p\left(\beta_{0},\beta_{1}.\sigma |D\right)=\frac{p\left(D|\beta_{0},\beta_{1}.\sigma \right)p\left(\beta_{0},\beta_{1}.\sigma \right)}{\int \int \int p\left(D|\beta_{0},\beta_{1}.\sigma \right)p\left(\beta_{0},\beta_{1}.\sigma \right)\text{d}\beta_{0}\text{d}\beta_{1}\text{d}\sigma }$$

The Bayesian framework aims to infer a likely distribution of the determined parameters *β*_0_, *β*_1_, and *σ* given the observed data *D*. The prior terms *p*(*β*_0_, *β*_1_.*σ*) at the denominator indicate the distribution of credibilities that the specific parameter could take without considering the observed data. The marginal likelihood *p*(*D*|*β*_0_, *β*_1_.*σ*)*p*(*β*_0_, *β*_1_.*σ*) informs us about the overall probability of the data *p*(*D*|*θ*), weighting these probabilities by the strength of their prior likelihood. All models conceive a hierarchical structure of the statistical analysis. That means, within the Bayesian framework, the first step is to apply a determined distribution that could best fit the dependent variable^5^.

All two models apply to the (log) CRP distribution, a t-student distribution. The choice of the t-student concerns the potential outliers present in the observed data, thus providing a more robust model.

Models 1 and 2 set the mean of the dependent variable as a linear function of the aforementioned covariates. The models exploit the hierarchical features of the Bayesian framework by calculating the deviations from the mean (as population-level effects) of the groups outlined by the two categorical variables representing individuals’ SES: occupation and education. The specification of Models 1 and 2 take the following form:

(2) $$\begin{array}{l} \mu_{y}=\beta_{0}+\sum_{i}\beta_{i}X_{i}+\beta_{j}X_{j}\\ p(\beta_{0})=N(\mu_{y},\sigma_{y})\\ p(\sigma_{\beta_{i}})=C(0,\sigma_{y}) \end{array}\;p(\beta_{j})\;=\;N(0,\;1)\;p(\beta_{i})\;=\;N(0,\;1)\;p(\sigma )\;=N(0,\;\sigma_{y})\;p(\nu )\;=\;e(1/29)\;$$

The prior distributions of Model 1 and Model 2 follow the suggestions from Gelman (2006) and Kruschke (2014). The hierarchical structure of Model 1 allows setting a prior distribution for the deviations from the mean of the SES categorical variables *p*(*β*_*i*_). Previous studies suggest that the prior distribution for the parameter *β*_*i*_ follows a (half-) Cauchy distribution with shape and scale parameters 0, *σ*_*y*_. The scale parameter at zero has a twofold function. As the prior distribution should inform the likely parameter regarding the deviations from the mean of the dependent variable of the SES measures occupation and education categories, the average deviation should be 0. Second, for efficiency reasons (i.e., the MCMC would not sample from implausible values), we wanted the values sampled from the MCMC to be not too far from the mean of the dependent variable. The prior distribution for the equivalized income (and the other continuous covariates) *p*(*β*_*j*_) informs model 1 about the more likely parameters (among all the possible in the hyperparameter space) for the slope coefficients. As we have centered the continuous variables as $X_{i}\left(c\right)=X_{i}-\bar{X_{i}}$, the prior distribution for the slopes is normally shaped with mean 0 and a standard deviation of 1. Finally, *p*(*ν*) is the exponentially distributed prior for the *ν* parameter of the t-student distribution. The *ν* parameter shapes the thickness of the tails of the distributions, thus accommodating the outliers.

Model 2 exploits the unique feature of the Bayesian framework, that is, the possibility to also model the variance across groups through distributional models. The difference between Model 1 and Model 2 is, thus, in Model 1, only the mean parameter can depend on predictors while *σ*_*y*_ is assumed to be constant across observations. Conversely, in Model 2, both *μ*_*y*_ and *σ*_*y*_ can be objective of the statistical modeling, thus relaxing the assumption of homogeneity of variance. Importantly, Model 2 identifies within-group differences in CRP distribution, i.e., estimating the differences in CRP for individuals at the same level of SES. The specification of Model 2 can be formalized as follows:

(3) $$\begin{array}{l} \mu_{y}\;\;\;\;\;=\;\beta_{0}+\sum_{i}\beta_{i}X_{i}+\beta_{j}X_{j}\\ ln(\sigma_{y})=\beta_{0}+\sum_{i}\beta_{i}X_{i}+\beta_{j}X_{j}\\ p(\sigma_{\sigma })\;=\;N(0,\;\sigma_{y}) \end{array}\;\begin{array}{l} p(\beta_{0})=N(\mu_{y},\sigma_{y})\\ p(\sigma_{\beta_{i}})=C(0,\sigma_{y}) \end{array}\;p(\beta_{j})\;=\;N(0,\;1)\;p(\beta_{i})\;=\;N(0,\;1)\;p(\sigma )\;=N(0,\;\sigma_{y})\;p(\nu )\;=\;e(1/29)\;$$

In Model 2, we also apply the same linear function for the log-transformed standard deviation of the dependent variable^6^. The two new prior distributions are *p*(*σ*_*σ*_) and $p\left(\sigma_{\beta_{i}}\right)$. The prior distribution *p*(*σ*_*σ*_) defines our expectations for the standard deviation of the *σ* distribution of the dependent variable. The prior $p\left(\sigma_{\beta_{i}}\right)$ defines the *a priori* distribution for the deviations relative to the categories of occupational status and education. The Bayesian Regression models were performed through R, using as a backend the Stan program. Specifically, Stan uses a particular algorithm of MCMC, defined as Hamiltonian Markov Chain (HMC)^7^.

We performed four MCMC chains that included 2000 iterations. We set the burn-in (i.e., the number of initial iterations not considered due to strong autocorrelation) as the first 1000 iterations (Kruschke, 2014).

## 3 Results

### 3.1 Model 1

**Fig. 1:**
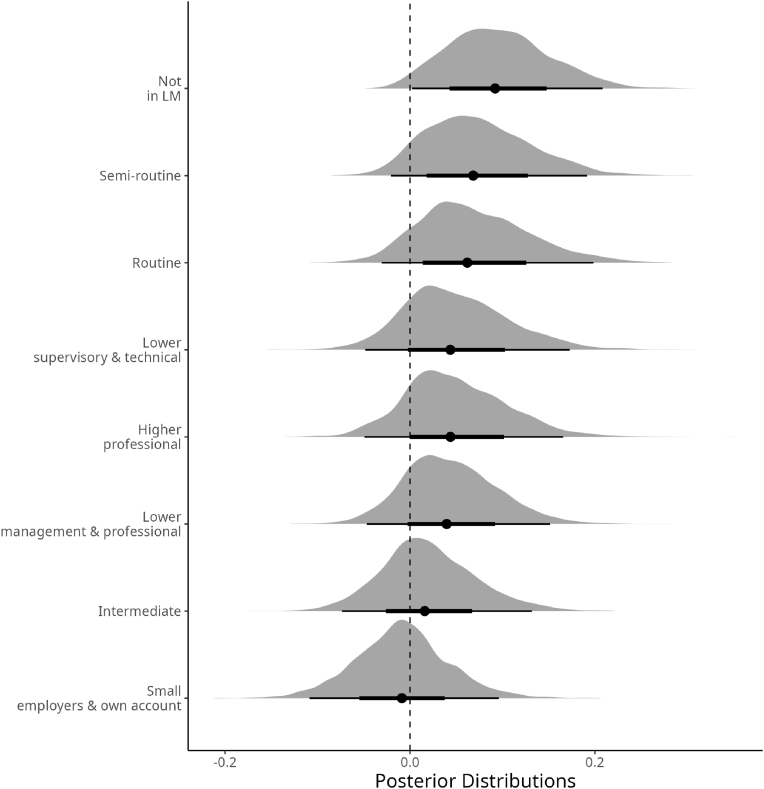
Posterior Distributions of the likely deviations$\sigma_{\beta_{i}}$ from the mean according to occupational status.

Table 3 shows the summary of the findings by Model 1. We focus on the results concerning the effects of occupation, education, and income on the distribution of log-CRP. Fig. 1 shows the posterior distributions of contrasts against the reference category (i.e., large employers & higher management) drawn by the MCMC for the concerns about occupational status. Under each distribution, the dot represents the expected value (i.e., the mean), while the thicker lines represent around 65% of the posteriors’ probability density function (PDF). The dashed black line serves as a reference for null-divergence of the distribution toward the grand mean of the dependent variable. Fig. 1 shows that small employers, in comparison with the other categories, have a lower concentration of CRP. Surprisingly, the lower categories of occupational status (lower supervisory and technical staff, semi-routine, routine, and individuals not in the labor market) do not show remarkable differences.

**Table 3:** Bayesian Regression results, assuming homogeneity of variance.

Parameter	*μ*	*σ*	2.5%	50%	97.5%
Intercept	0.379	0.060	0.262	0.378	0.499
**Gender: Female**					
Gender (Female)	0.150	0.021	0.110	0.150	0.194
**House Ownership: Owner**					
Rent	0.062	0.027	0.009	0.062	0.116
Other	0.063	0.089	−0.109	0.062	0.240
**SRH: Excellent**					
Very good	0.079	0.031	0.019	0.079	0.137
Good	0.084	0.033	0.018	0.084	0.146
Fair	0.198	0.039	0.120	0.198	0.273
Poor	0.333	0.056	0.226	0.333	0.436
**Smoking: Smoker**					
Ex smoker	−0.221	0.030	−0.282	−0.221	−0.161
Non smoker	−0.257	0.030	−0.317	−0.257	−0.199
Sport Activity	−0.075	0.012	−0.098	−0.075	−0.052
Age	0.085	0.013	0.058	0.085	0.111
Income	−0.015	0.011	−0.036	−0.015	0.006
BMI	0.386	0.010	0.366	0.386	0.407
Std Education (Intercept)	0.087	0.056	0.030	0.073	0.222

Std Occupation (Intercept)	0.055	0.027	0.015	0.050	0.121
*σ*	0.876	0.011	0.855	0.876	0.898
*ν*	9.747	0.891	8.220	9.688	11.766
**Education, Intercepts**					
Degree	−0.057	0.046	−0.155	−0.054	0.023
Other higher degree	−0.038	0.048	−0.141	−0.035	0.046
A-level	0.003	0.046	−0.094	0.005	0.090
GCSE	−0.034	0.045	−0.134	−0.031	0.048
Other qualification	0.038	0.048	−0.058	0.038	0.131
No qualification	0.086	0.048	−0.005	0.084	0.182
**Occupation, Intercepts**					
Large employers.& higher management	−0.043	0.046	−0.146	−0.039	0.034
Higher professional	0.006	0.038	−0.069	0.005	0.085
Lower management & professional	0.0001	0.031	−0.060	−0.000	0.063
Intermediate	−0.023	0.034	−0.096	−0.022	0.041
Small employers & own account	−0.052	0.040	−0.138	−0.048	0.014
Lower supervisory &.technical	0.007	0.038	−0.065	0.005	0.085
Semi-routine	0.030	0.035	−0.031	0.028	0.103
Routine	0.026	0.038	−0.043	0.023	0.107
Not in LM	0.052	0.031	−0.002	0.051	0.120

**Fig. 2:**
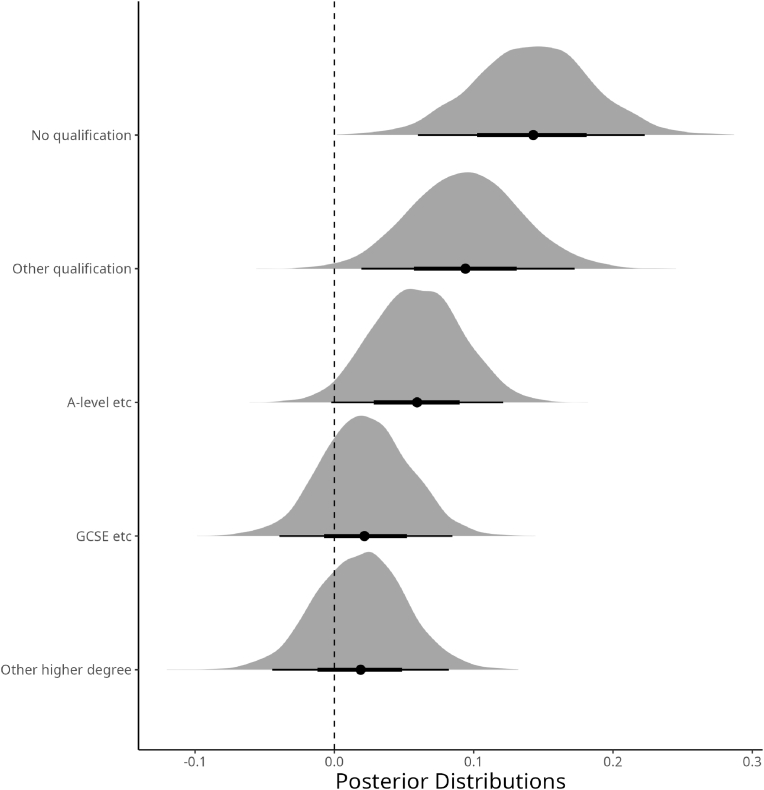
Posterior Distributions of the likely deviations from the mean according to Educational levels. $\sigma_{\beta_{i}}$.

Regarding the educational level and CRP concentration, the second SES measure shows a higher level of CRP for individuals with no educational qualification concerning the referent category (i.e., individuals with educational degrees), as depicted in Fig. 2. Conversely, individuals with higher educational degrees (e.g., with GCSE and higher degrees) are more likely to have lower CRP concentrations in the blood.

**Fig. 3:**
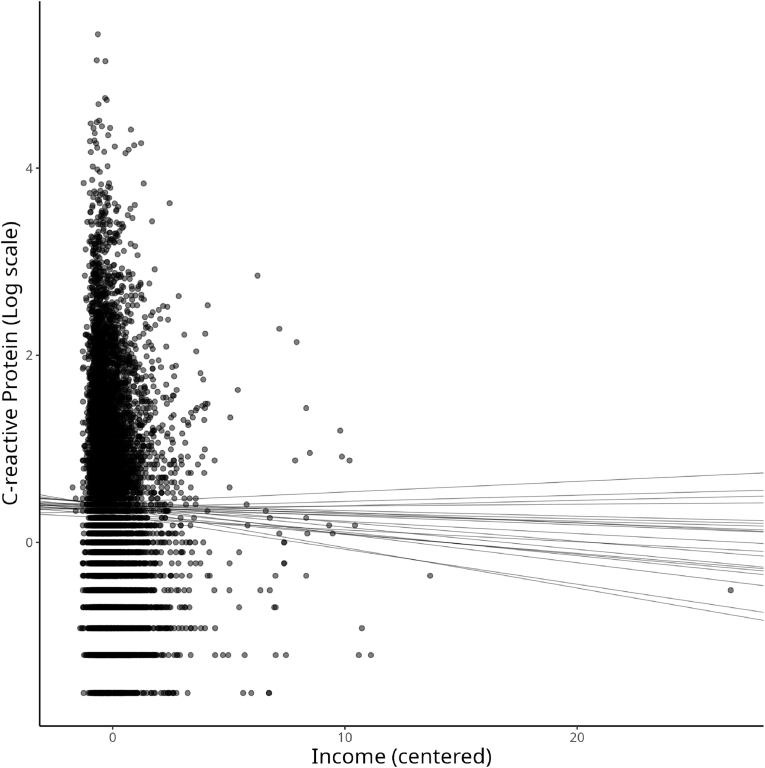
Plot of the income distribution (on the *x*-axis) and log-CRP (on the *y*-axis) and model fit of 20 possible regression lines sampled from the posterior distribution *β* Income of Model 1.

To depict the relationship between household income (equivalized) and levels of CRP, we make use of scatterplots where the *x*-axis represents the levels of household income (in the centered scale) and the levels of (log) CRP on the *y*-axis. Fig. 3 shows this relationship. Conversely, to the traditional frequentist approach, it is possible to visualize different likely regression lines in the Bayesian framework, as the MCMC samples from the posterior distribution of likely regression slopes. In Fig. 3, we show 20 possible regression lines assessing the relationship between income and CRP.

From Fig. 3, it is possible to recognize a general negative relationship between equivalized income and levels of CRP. Thus, economic resources could slightly impact the risks of CVDs measured through CRP. However, the slopes of the regression lines are not relatively steep, suggesting a mild negative relationship of −0.02 (i.e., the mean of the income posterior distribution presented in Table 3). From Fig. 3, it seems that Model 1 is affected by the leverage effect.

### 3.2 Model 2

In Model 2, we relaxed the homogeneity of variance assumption among the categories of occupational status and educational levels, thus predicting the mean of the dependent variable *μ* and its standard deviation *σ*. This subsection shows the results from modeling the *σ* parameter. As in the previous section, we first provide a tabular format of the results provided by Model 2 as in Table 4. The table makes it possible to find the results in modeling the parameter *μ* and the *σ* parameters.

**Table 4:** Bayesian Regression results, relaxing the assumption of homogeneity of variance.

Parameter	*μ*	*σ*	2.5%	50%	97.5%
Intercept	0.370	0.066	0.253	0.372	0.485
*σ* Intercept	−0.155	0.041	−0.235	−0.155	−0.075
**Gender: Female**					
Gender (female)	0.159	0.020	0.118	0.159	0.197
*σ* Gender (female)	0.022	0.018	−0.013	0.022	0.057
**House Ownership: Owner**					
Rent	0.059	0.028	0.005	0.059	0.112
Other	0.058	0.091	−0.118	0.058	0.229
*σ*Rent	0.012	0.023	−0.031	0.012	0.057
*σ*Other	0.008	0.080	−0.142	0.006	0.167
**SRH: Excellent**					
Very good	0.083	0.030	0.025	0.082	0.142
Good	0.090	0.032	0.026	0.090	0.152
Fair	0.203	0.039	0.126	0.204	0.279
Poor	0.338	0.057	0.227	0.337	0.450
*σ*Very good	0.047	0.027	−0.004	0.048	0.099
*σ*Good	0.056	0.028	0.001	0.056	0.114
*σ*Fair	0.095	0.033	0.031	0.094	0.161
*σ*Poor	0.064	0.047	−0.026	0.062	0.155
**Smoking: Smoker**					
Ex smoker	−0.220	0.031	−0.280	−0.220	−0.161
Non smoker	−0.261	0.031	−0.320	−0.261	−0.199
*σ*Ex smoker	−0.058	0.025	−0.107	−0.058	−0.009
*σ*Non smoker	−0.089	0.026	−0.138	−0.089	−0.040
Sport Activity	−0.058	0.025	−0.107	−0.058	−0.009
*σ*Sport Activity	−0.089	0.026	−0.138	−0.089	−0.040
Age	0.080	0.014	0.053	0.080	0.106
*σ* Age	−0.027	0.011	−0.049	−0.027	−0.005
Income	−0.015	0.011	−0.036	−0.015	0.007
*σ* Income	−0.004	0.010	−0.023	−0.004	0.015
BMI	0.386	0.011	0.365	0.385	0.407
*σ*BMI	−0.040	0.009	−0.057	−0.039	−0.022
Std Education (Intercept)	0.079	0.053	0.026	0.067	0.205
Std Occupation (Intercept)	0.057	0.027	0.019	0.053	0.124
*σ*Std Education (Intercept)	0.024	0.020	0.001	0.020	0.077
*σ*Std Occupation (Intercept)	0.052	0.024	0.021	0.048	0.111
*ν*	10.357	1.044	8.552	10.272	12.606
**Education Intercepts**					
Degree	−0.052	0.052	−0.142	−0.051	0.030

Other higher degree	−0.032	0.052	−0.126	−0.032	0.051
A-level	0.006	0.052	−0.083	0.004	0.096
GCSE	−0.029	0.051	−0.114	−0.029	0.055
Other qualification	0.034	0.055	−0.053	0.031	0.134
No qualification	0.080	0.056	−0.006	0.075	0.184
**Occupation Intercepts**					
Large employers & higher management	−0.043	0.045	−0.142	−0.039	0.033
Higher.professional	0.004	0.040	−0.075	0.003	0.088
Lower management & professional	−0.000	0.030	−0.060	−0.001	0.061
Intermediate	−0.024	0.035	−0.098	−0.022	0.042
Small employers & own account	−0.056	0.038	−0.137	−0.054	0.010
Lower supervisory & technical	0.007	0.040	−0.071	0.005	0.090
Semi-routine	0.029	0.036	−0.036	0.026	0.105
Routine	0.027	0.039	−0.043	0.024	0.109
Not in LM	0.058	0.032	0.000	0.056	0.125
***σ*Education, Intercepts**					
Degree	−0.006	0.018	−0.046	−0.003	0.027
Other higher degreee	−0.007	0.019	−0.051	−0.003	0.027
A-level	0.007	0.018	−0.026	0.005	0.047
GCSE	−0.015	0.020	−0.062	−0.011	0.016

Other qualification	0.017	0.022	−0.014	0.012	0.070
No qualification	0.004	0.019	−0.033	0.002	0.047
***σ*Occupation, Intercepts**					
Large employers.&.higher management	−0.020	0.037	−0.096	−0.018	0.048
Higher professional	0.037	0.037	−0.027	0.034	0.116
Lower management & professional	−0.028	0.028	−0.085	−0.027	0.026
Intermediate	−0.007	0.032	−0.068	−0.007	0.058
Small employers & own account	−0.057	0.038	−0.136	−0.055	0.009
Lower supervisory & technical	0.011	0.035	−0.055	0.010	0.083
Semi-routine	0.016	0.031	−0.042	0.015	0.082
Routine	−0.009	0.034	−0.076	−0.009	0.055
Not in LM	0.057	0.027	0.008	0.056	0.115

**Fig. 4:**
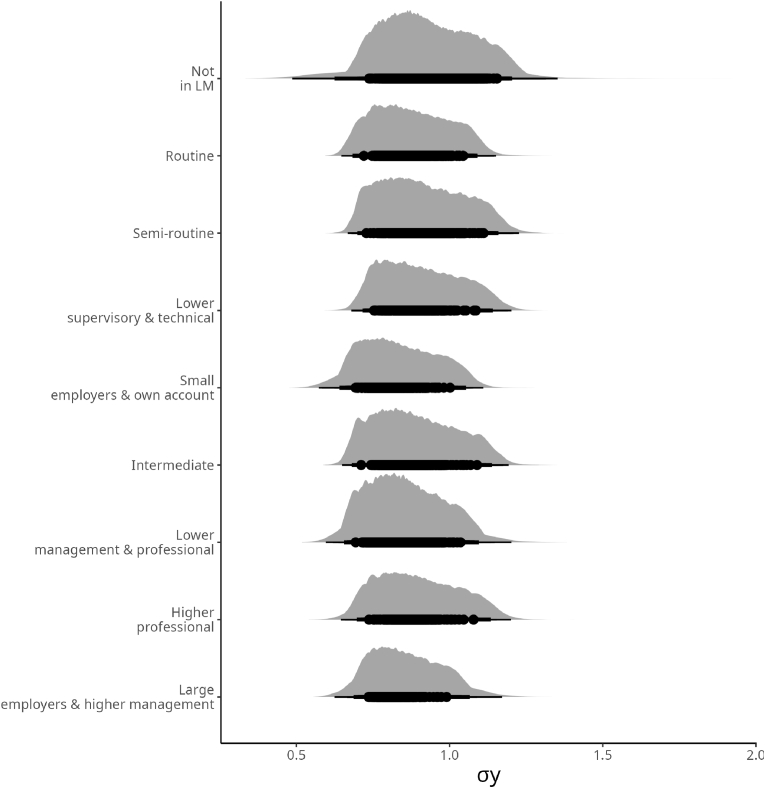
Posterior Distributions of the likely deviations from the mean according to Occupational status. *σ* occupation on the *σ*_*y*_.

Here, we focus on the results of fitting the model in the *σ* of the dependent variable. Similar to the section dedicated to the results of Model 1, we started by presenting the posterior distributions related to the individuals’ occupational status.Fig. 4 shows the posterior distributions for each category of occupational status and how they deviate according to the scale of the standard deviation of the CRP levels.

When the model relaxes the assumption of homogeneity of variance, the findings suggest a rather clear variance between the large employers and higher management category and individuals not in the labor market. However, the in-between categories show a similar pattern.

**Fig. 5:**
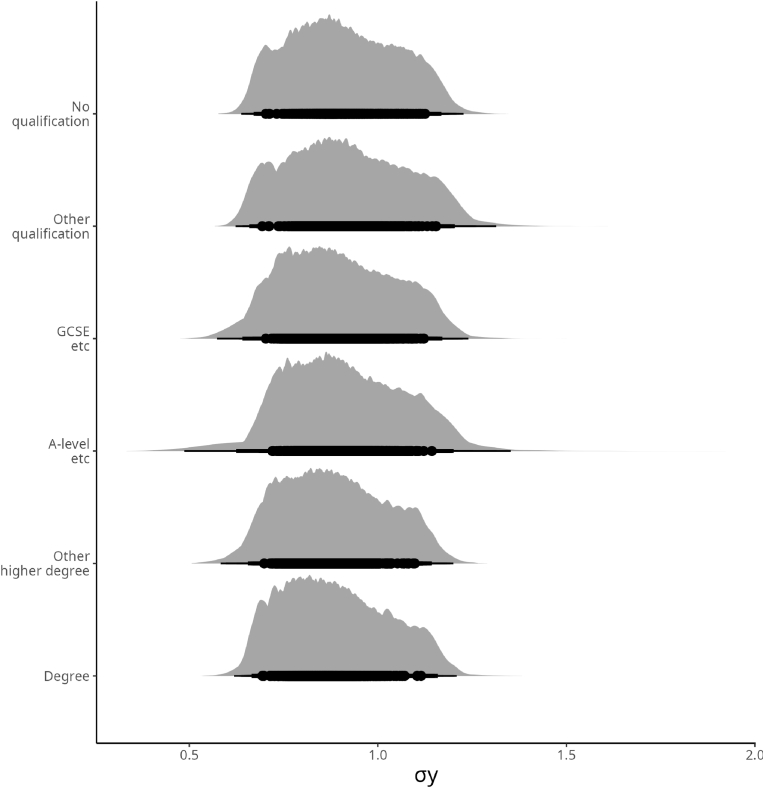
Posterior Distributions of the likely deviations from the mean according to Educational levels. *σ* Education on the *σ*_*y*_.

Moving to the effects of educational status on the variance between categories on the variance of CRP observed through the data, Fig. 5 shows the findings of Model 2 focused on individuals’ education.

From Fig. 5, the findings of Model 2 show a surprising similarity between the educational levels, with the notable exception of the individuals with an A-level. In fact, from Model 2, the variance for the individuals with an A-level educational degree is stretched toward the right.

**Fig. 6:**
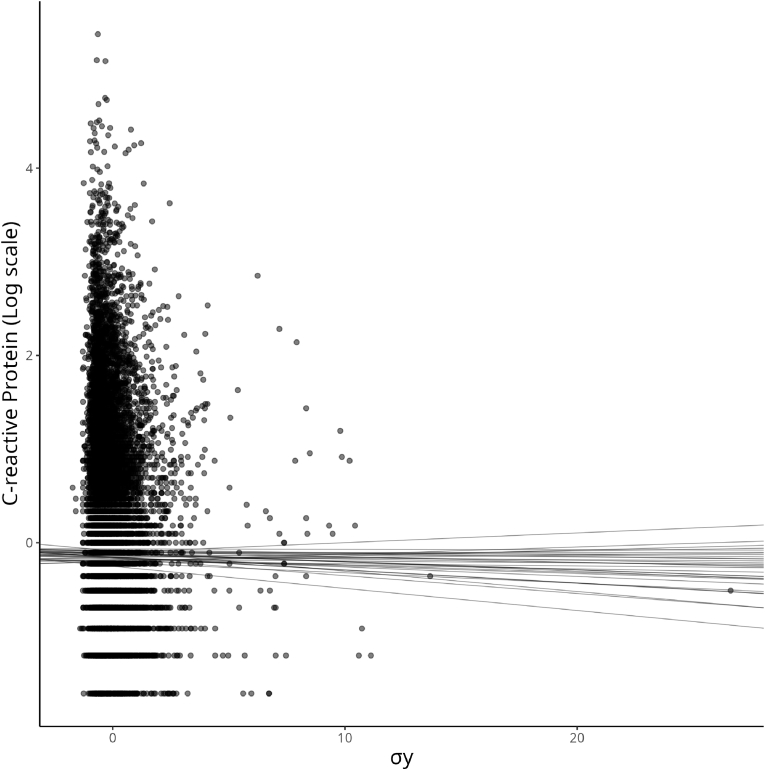
Plot of the income distribution (on the *x*-axis) and log-CRP (on the *y*-axis) and model fit of 20 possible regression lines sampled from the posterior distribution *β* Income of Model 2.

Similar to Model 1, to present the findings from Model 2 concerning the relationship between equivalized income and logged CRP, Fig. 6 depicts 20 sampled regression lines from the posterior distribution of the regression slopes. However, the intercepts and slopes are computed according to *σ*_*y*_ in this case. From Fig. 6, findings suggested an even weaker relationship between equivalized income and logged CRP, with the model’s homogeneity of variance assumption relaxed.

In the Supplementary Materials (Appendix A), we provide the trace and the autocorrelation plots. These are valuable tools to assess the Bayesian inference’s validity and the algorithm’s correct sampling from the posterior distributions of the specific parameters.

## 4 Discussion & conclusions

A main current challenge in social stratification and health inequalities is the assessment of the mechanisms through which the well-documented link between deprived social conditions and health worsening takes form. The rising availability of social data that includes biological information about individuals is rising as a propitious track of research, and it has a possible double effect. First, social scientists acquire additional information potentially helpful to studying the mechanisms through which the social gradient of health occurs and socioeconomic status gets under the skin. Second, sociologists inform the biomedical literature on the importance of individuals’ social and economic environments for health levels. This study investigated one of the potential mechanisms that health inequalities generate. Indeed, this study aimed to shed light on the connection between socioeconomic status (SES), and levels of low-grade systemic inflammation, measured through C-reactive protein (CRP). Biomedical evidence have linked high levels of CRP to increased mortality risks due to cardiovascular diseases (CVDs). We consider this pathway particularly important as mortality due to CVDs represents the first cause of death among individuals in developed countries (Brummett et al., 2014). We used data from the Understanding Society - the United Kingdom Household Longitudinal Study (UKHLS), a representative sample of the population living in England, Scotland, and Wales. In 2012, the UKHLS collected blood samples from voluntary individuals to collect markers of socially relevant health risk factors and diseases. To capture the multifaceted characteristics of individuals’ SES, we included three measures of social conditions in the statistical analysis: occupational status, educational levels, and equivalized household income. This study provides a Bayesian framework to assess the pathway that links individuals’ SES, mortality risks, and levels of CRP. The first Bayesian regression model (BRM) provides posterior distributions of likely effect magnitude parameters assuming homogeneity of variance across occupational and educational groups. The second step of the statistical analysis deploys a distributional model, relaxing the assumption of homogeneity of variance through modeling the standard deviation of CRP along its mean. Generally, all models show no inference problems, meaning the posterior distributions computed by both models represent all possible distributions. The concern of the findings of Model 1 is that the educational gradient is the most vital determinant of the risk of low-grade inflammation, while equivalized income is the weakest among the three SES measures. According to Davillas et al. (2017), one potential explanation is that better-educated individuals tend to pursue a healthier lifestyle and be more aware of health risks. The relationship between occupational status and levels of CRP seems to be polarized. Indeed, the findings suggest a homogeneity of CRP levels and lowest levels between individuals with the highest occupational status and small employers. That means they do not deviate remarkably from the grand mean of the dependent variable among all the other categories. While the first findings are coherent with previous literature (Marmot et al., 1991), we strongly suggest that future research deepen scientific knowledge of small employers’ concerns. Regarding the equivalized income measure, some lessons can be inferred from both the methodological and substantial perspectives: the methodological conclusion is that even in the Bayesian framework, the regression model could suffer from leverage effects. The significant lesson is that economic resources are the weakest social determinant of CRP.

The findings presented in the previous section suggest that health-related behaviors are important in shaping the relationship between SES and CRP. In fact, the posterior distributions obtained by the BRM concerning smoking behavior show a significant increase in CRP levels for smokers compared with ex- and nonsmokers and a strong impact of BMI on CRP. The harmful effect of BMI is consistent within SES categories, as indicated by the low variance coefficients (see Model 2).

Model 2 provides exciting results for how individuals differ within each category of the SES measures. Indeed, the findings suggest a strong cohesion in the highest class of occupational status, meaning that inequalities in health are evident even when we consider the within-variance. Surprisingly, individuals with the same educational level are not very dissimilar, except for individuals with an A-level degree.

It is worth noting that the meaning of income may change during the different stages of the life course we considered in the analysis. Therefore, we suggest investigating this aspect further by, for example, considering a more homogenous sample in terms of age.

This study has some limitations that future researchers should consider. The first limitation is the lack of direct comparison between the posterior distributions explicitly drawn for income, education, and occupational status categories. Future research could address this problem ideally from a Bayesian perspective. The second limitation concerns the model specification. Due to the already complex Bayesian models, we were unable to test the models for nonlinearities in age patterns and CVD risks through CRP. We believe it could be interesting to see whether the aging process could take other, more complex ways. The third limitation concerns the omission of other potential explanatory factors in the regression model, such as the presence of comorbidities, family’s disease history, the type of individuals’ diet, their living location, and their ethnicity. Future research could expand the dimensions of the regression model by including these relevant factors.

Furthermore, the study’s findings are based on data from 2012. While certain conclusions may retain their validity, more recent data sources would enable an assessment of the potential impact of the COVID-19 pandemic. The final limitation concerns robustness checks. Indeed, we specified the models only with this set of prior distributions. Hence, we invite the social scientific community to use our results for better-refined models coherently with the Bayesian philosophy. The Understanding Society data provides the empirical researcher with a wide range of biological markers of (ab-) normal physiological functioning. Future research could potentially exploit the biological information collected by empirically testing the social gradient of health using composite measures such as Allostatic Load. Future researchers could take advantage of this first Bayesian implementation and use the results we provided as a starting point to define a theoretically guided model to advance our knowledge concerning the social gradient of mortality risks.

## Author statement

Procopio Alessandro: Conceptualization, Formal Analysis, Methodology, Writing –Original Draft, Robin Samuel: Conceptualization, Validation, Writing – Review & Editing, Supervision, Planning.

## Declaration of competing interest

The authors declare that they have no known competing financial interests or personal relationships that could have appeared to influence the work reported in this paper.

## Figures and Tables

**Fig. 7:**
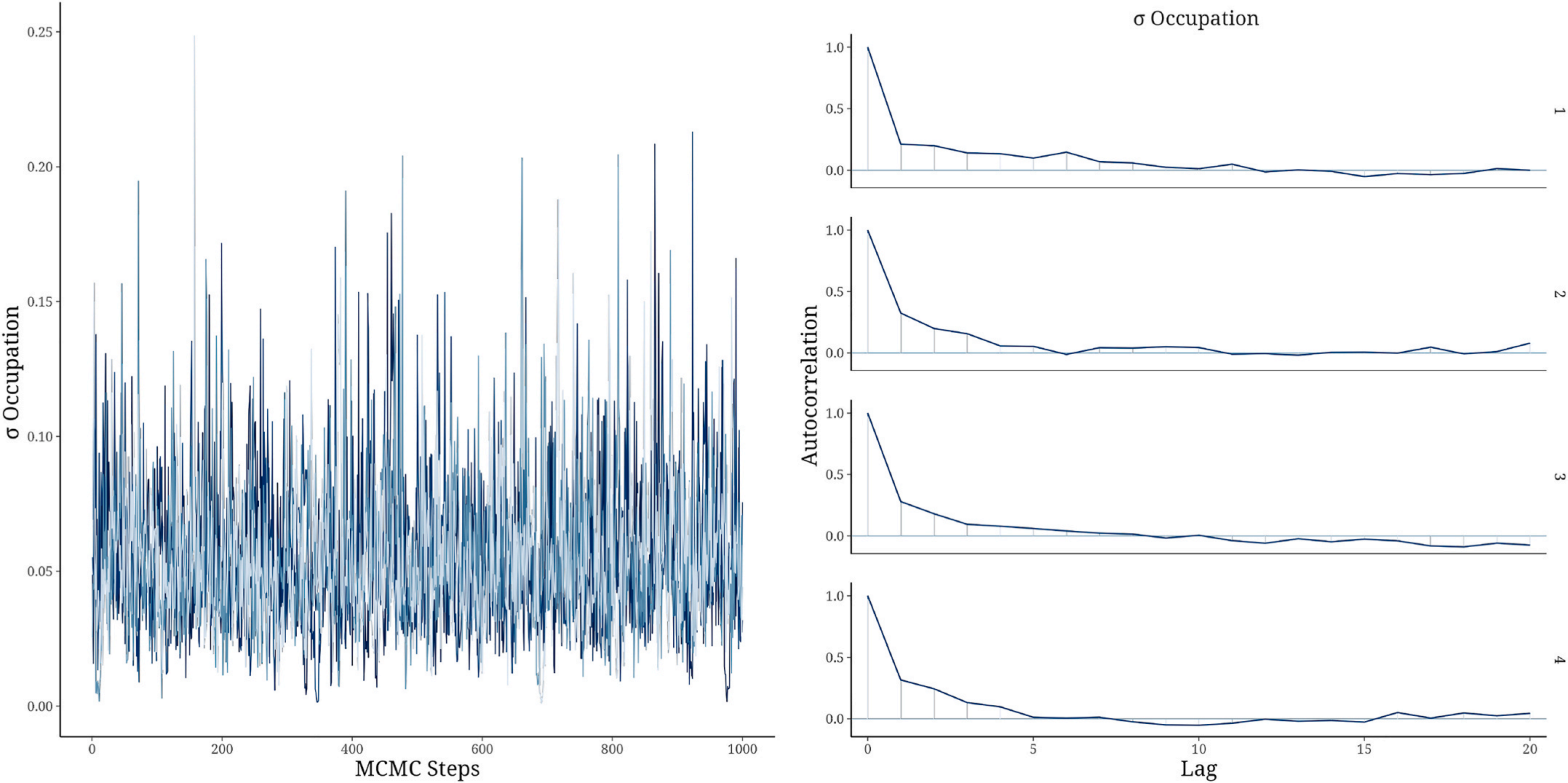
Panel (a): Trace plot of the occupational status parameter $\sigma_{\beta_{i}}$. Panel (b): Autocorrelation plot of $\sigma_{\beta_{i}}$ by the number of chains of the MCMC..

**Fig. 8:**
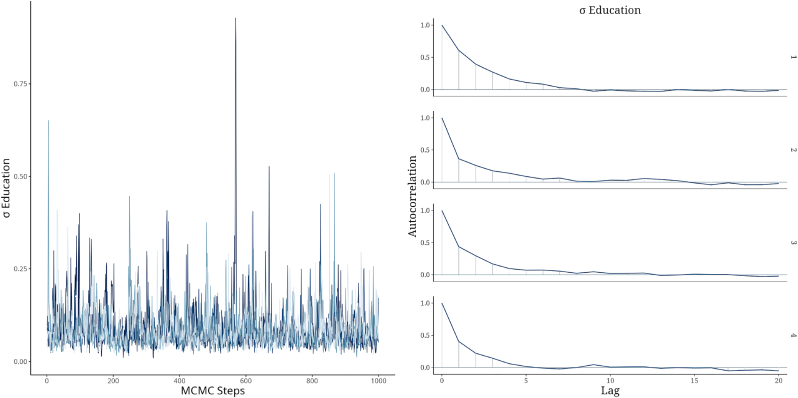
Panel (a): Trace plot of the educational attainment parameter $\sigma_{\beta_{i}}$. Panel (b): Autocorrelation plot of $\sigma_{\beta_{i}}$ by the number of chains of the MCMC..

**Fig. 9:**
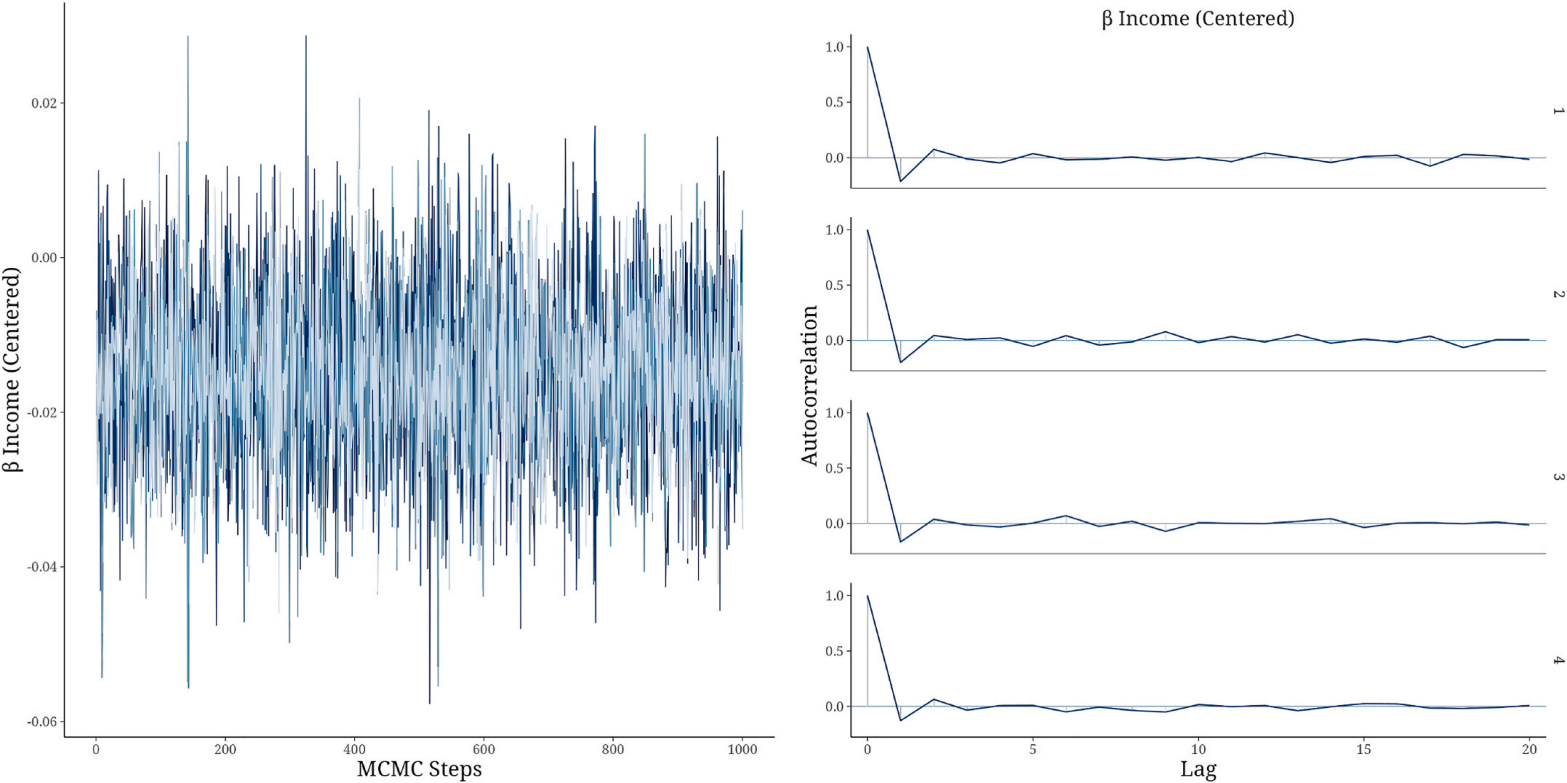
Panel (a): Trace plot of the *β* income parameter. Panel (b): Autocorrelation plot of *β* by the number of chains of the MCMC. Model 2.

**Fig. 10:**
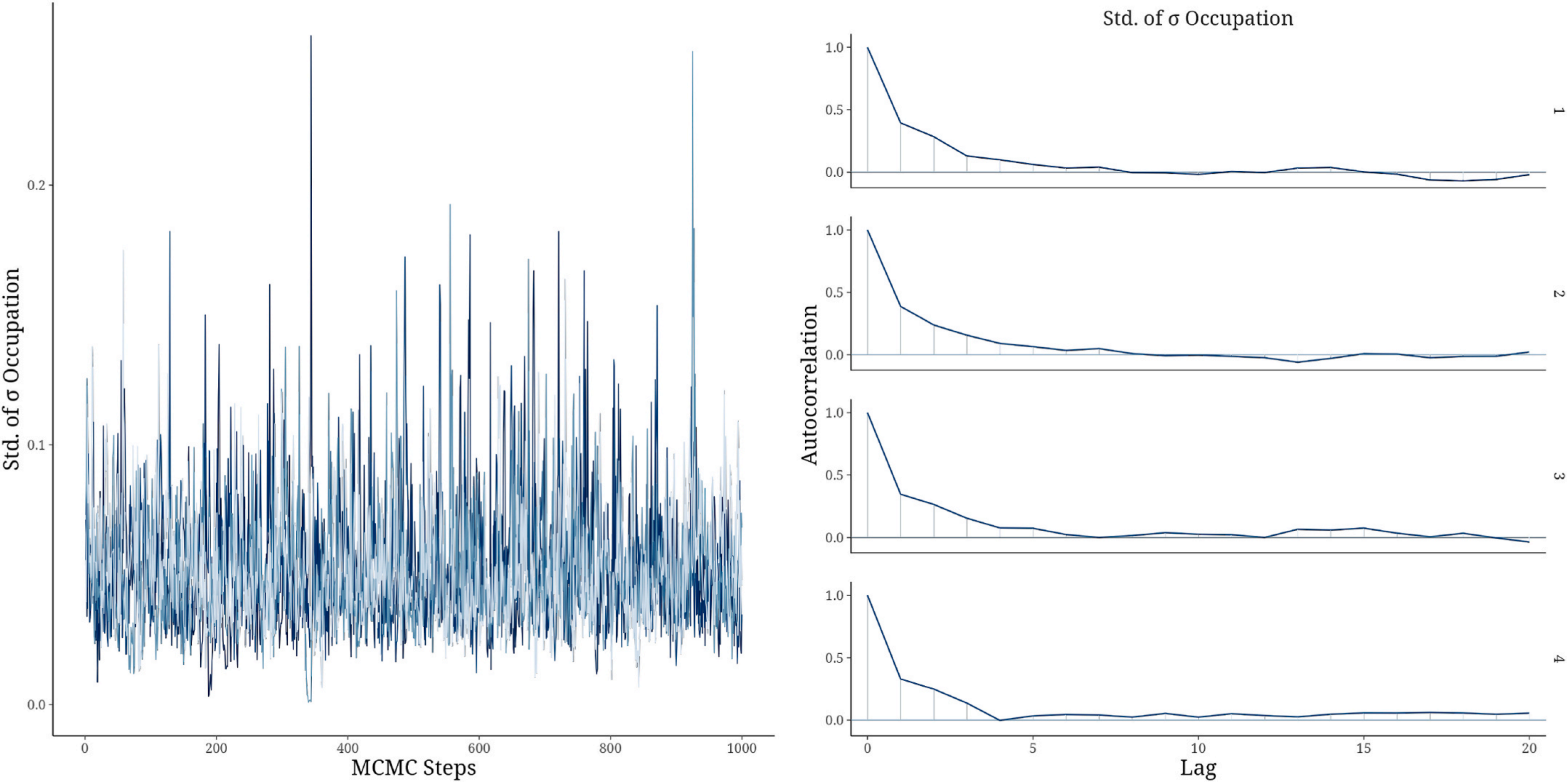
Panel (a): Trace plot of the occupational status parameter $\sigma_{\beta_{i}}$ on *σ*_*y*_. Panel (b): Autocorrelation plot of $\sigma_{\beta_{i}}$ by the number of chains of the MCMC..

**Fig. 11:**
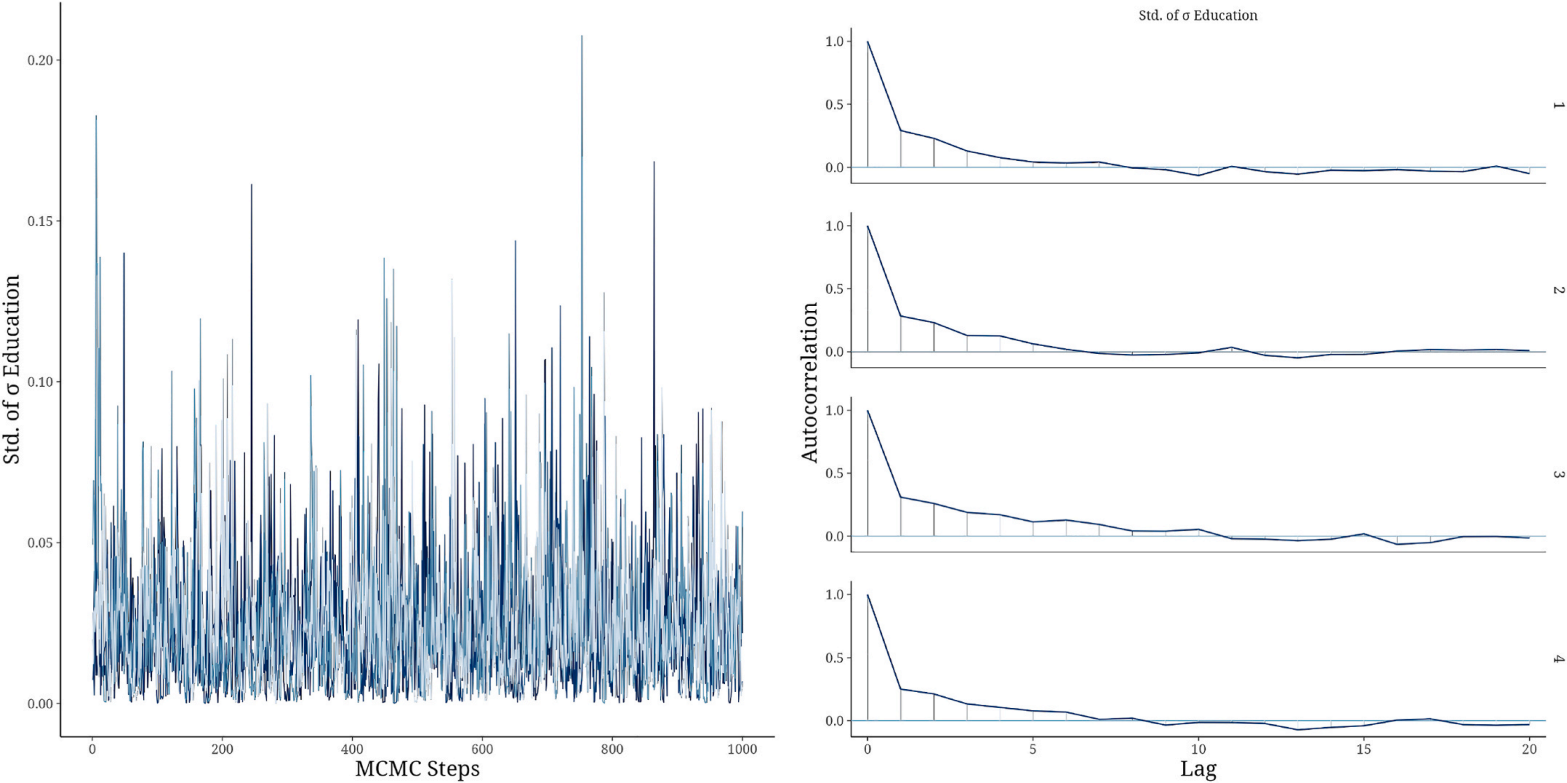
Panel (a): Trace plot of the educational attainment parameter $\sigma_{\beta_{i}}$ on *σ*_*y*_. Panel (b): Autocorrelation plot of $\sigma_{\beta_{i}}$ by the number of chains of the MCMC..

**Fig. 12:**
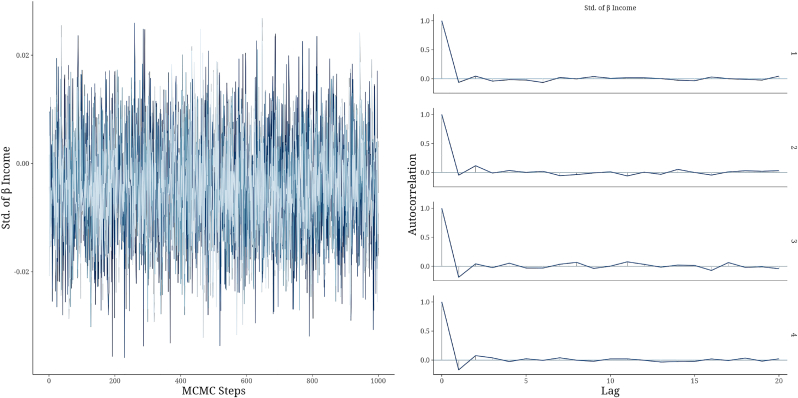
Panel (a): Trace plot of the *β* income parameter on *σ*_*y*_. Panel (b): Autocorrelation plot of *β* by the number of chains of the MCMC..

**Table 5:** Summary statistics of the covariates used in the analysis by missing indicator variables on CRP, BMI, and on the covariates. Table 1 reports the percentages and proportion confidence intervals for the categorical variables. For the continuous variables, Table 1 reports the mean; confidence intervals of the mean are in parentheses.

	Missing values on CRP (N = 521)	Missing values on BMI (N = 283)	Missing Values overall (N = 936)	Analytical Sample (N = 8960)
**Occupation (%)**				
Large employers & higher management	3.07	1.06	2.32	3.13
	(0.018, 0.049)	(0.002, 0.031)	(0.014, 0.035)	(0.028, 0.035)
Higher professional	5.56	3.91	4.76	4.42
	(0.038, 0.079)	(0.020, 0.069)	(0.035, 0.064)	(0.040, 0.048)
Lower management & professional	17.27	10.32	13.62	16.00
	(0.141, 0.208)	(0.070, 0.145)	(0.115, 0.160)	(0.153, 0.167)
Intermediate	8.06	5.33	6.53	7.74
	(0.059, 0.107)	(0.030, 0.087)	(0.050, 0.083)	(0.072, 0.083)
Small employers & own account	6.14	7.11	5.30	5.54
	(0.042, 0.086)	(0.044, 0.108)	(0.044, 0.076)	(0.051, 0.060)
Lower supervisory & technical	4.03	2.49	3.32	4.25
	(0.025, 0.061)	(0.010, 0.051)	(0.023, 0.047)	(0.039, 0.047)
Semi-routine	8.44	7.47	8.30	9.34
	(0.062, 0.112)	(0.047, 0.112)	(0.066, 0.103)	(0.088, 0.099)
Routine	6.52	4.27	6.20	5.54
	(0.046, 0.090)	(0.022, 0.073)	(0.047, 0.080)	(0.051, 0.060)
Not in LM	40.88	58.0	49.05	43.99
	(0.366, 0.452)	(0.520, 0.638)	(0.458, 0.524)	(0.430, 0.450)
**Education (%)**				
Degree	28.51	17.43	25.754	22.41
	(0.246, 0.327)	(0.132, 0.224)	(0.229, 0.288)	(0.216, 0.233)
Other higher degree	12.47	10.32	11.48	13.50
	(0.097, 0.157)	(0.070, 0.145)	(0.094, 0.138)	(0.128, 0.142)
A-level etc	20.0	14.23	17.51	18.17
	(0.166, 0.238)	(0.104, 0.189)	(0.150, 0.202)	(0.174, 0.190)
GCSE etc	18.21	21.70	19.83	19.70
	(0.149, 0.219)	(0.170, 0.270)	(0.172, 0.227)	(0.189, 0.205)
Other qualification	11.28	11.74	10.78	11.65
	(0.087, 0.144)	(0.082, 0.161)	(0.088, 0.131)	(0.110, 0.123)
No qualification	9.50	24.55	14.61	14.54
	(0.071, 0.124)	(0.196, 0.300)	(0.123, 0.172)	(0.139, 0.153)
**Gender (%)**				
Male	46.25	48.41	47.32	44.44
	(0.419, 0.506)	(0.425, 0.544)	(0.441, 0.506)	(0.435, 0.454)
Female	53.74	51.59	52.67	55.55
	(0.494, 0.581)	(0.456, 0.575)	(0.494, 0.559)	(0.546, 0.565)
**House Ownership (%)**				
Owned	74.51	62.89	69.89	75.63
	(0.705, 0.782)	(0.570, 0.685)	(0.668, 0.729)	(0.748, 0.765)
Rent	23.55	35.33	28.35	23.06
	(0.200, 0.274)	(0.298, 0.412)	(0.254, 0.314)	(0.222, 0.239)
Other	1.93	1.76	1.75	1.30
	(0.009, 0.035)	(0.006, 0.041)	(0.010, 0.028)	(0.011, 0.016)
**Self-rated Health (%)**				
Excellent	21.30	7.77	18.15	15.67
	(0.179, 0.251)	(0.049, 0.115)	(0.157, 0.208)	(0.150, 0.164)
Very good	38.38	21.55	32.65	35.21
	(0.342, 0.427)	(0.169, 0.268)	(0.296, 0.358)	(0.343, 0.362)
Good	22.07	0.269	24.16	28.27
	(0.186, 0.259)	(0.218, 0.324)	(0.214, 0.271)	(0.274, 0.292)
Fair	12.28	26.85	16.21	15.43
	(0.096, 0.154)	(0.208, 0.313)	(00.139, 0.187)	(0.147, 0.162)
Poor	5.95	18.02	8.80	5.39
	(0.041, 0.083)	(0.137, 0.230)	(0.071, 0.108)	(0.050, 0.059)
**Smoking (%)**				
Smoker	18.81	19.08	18.18	19.53
	(0.155, 0.224)	(0.147, 0.242)	(0.158, 0.208)	(0.188, 0.203)
Ex-smoker	36.27	45.23	37.75	40.09
	(0.321, 0.406)	(0.393, 0.512)	(0.346, 0.409)	(0.391, 0.411)
Non-smoker	44.91	35.68	44.06	0.404
	(0.406, 0.493)	(0.301, 0.416)	(0.409, 0.473)	(0.394, 0.413)
**Sport Activity**				
Mean (CI)	3.988	2.198	3.543	3.412
	(3.725, 4.252)	(1.889, 2.507)	(3.346, 3.740)	(3.355, 3.469)
**Age**				
Mean (CI)	45.965	56.360	47.349	51.622
	(44.390, 47.541)	(54.209, 58.512)	(46.096, 48.603)	(51.286, 51.958)
**Equivalized Income**				
Mean (CI)	2.145.61	1.708.57	1.975.45	1.978.41
	(1977.29, 313.94)	(1539.69, 1877.46)	(1863.66, 2087.23)	(1947.77, 2010.05)
**BMI**				
Mean (CI)	25.144	27.783		
	(24.670, 25.618)	(27.680, 27.886)		

**Table 6:** Results of subgroup analysis concerning missing values, compared to analytical sample. Categorical variables report the Chi-squared association statistic value. Continuous variables report the t-student statistic value. Degrees of freedom are in parentheses.

	Missing values on CRP (N = 521)	Missing values on BMI (N = 283)	Missing Values overall (N = 936)
Occupation	5.32	30.38***	17.71*
	(8)	(8)	(8)
Education	20.18***	28.34***	8.37
	(5)	(5)	(5)
Gender	0.65819	16.957	3.36
	(1)	(1)	(1)
House Ownership	1.77	25.76***	18.10
	(2)	(2)	(2)
SRH	24.06***	133.45***	34.62***
	(4)	(4)	(4)
Smoking Behavior	4.95	3.53	5.85***
	(2)	(2)	(2)
Age	7.28***	−4.41***	7.12***
	(570.55)	(296.36)	(1082.8)
Sport Activity	−4.43***	7.83***	−1.37
	(573.1)	(302.6)	(1110.6)
Equivalized Income	−2.03*	3.18***	0.05
	(555.9)	(300.58)	(1089.5)
BMI	11.28***		
	(551.27)		

## Data Availability

Data will be made available on request.

## Footnotes

2 The UKHLS is a continuation and further evolution of the older British Household Panel Study (BHPS).

3 Alongside the GPS, the main survey target sample consisted of three additional components: the Ethnic Minority Boost sample, the former BHPS sample, and the Immigrant and Ethnic Minority Boost sample.

4 We conducted a subgroup analysis by testing the difference in observable values of the covariates included in the model. Appendix B provides a summary statistics table on the characteristics of the sample having missing values and results of statistical significance tests of difference between the analytical sample and the individuals with missing values on covariates included in models 1 and 2.

5 The choice of the distribution is dependent upon the type of variable the outcome: for instance, the Poisson distribution applies with count data; for instance, if the analysis aims to model a dichotomous variable, then the Bayesian framework will start with a binomial distribution.

6 We have transformed the standard deviation as the Stan Development Team recommended.

7 More precisely, Stan’s specification of the HMC is the No-U-Turn Shape (NUTS) algorithm.
